# A Developmental and Sequenced One-to-One Educational Intervention for Autism Spectrum Disorder: A Randomized Single-Blind Controlled Trial

**DOI:** 10.3389/fped.2016.00099

**Published:** 2016-09-26

**Authors:** Antoine Tanet, Annik Hubert-Barthelemy, Graciela C. Crespin, Nicolas Bodeau, David Cohen, Catherine Saint-Georges, The GPIS Study Group, Véronique Bur

**Affiliations:** ^1^Institut des Systèmes Intelligents et de Robotiques, Université Pierre et Marie Curie, Paris, France; ^2^Departement de Psychiatrie de l’Enfant et de l’Adolescent, APHP, Groupe Hospitalier Pitié-Salpêtrière et Université Pierre et Marie Curie, Paris, France; ^3^Croix Rouge Française, Paris, France; ^4^Association Programme de Recherche et d’Etudes sur l’Autisme, Paris, France; ^5^Hôpital de jour Centre André Boulloche, Paris, France

**Keywords:** autism, intellectual disability, randomized controlled trial

## Abstract

**Introduction:**

Individuals with autism spectrum disorder (ASD) who also exhibit severe-to-moderate ranges of intellectual disability (ID) still face many challenges (i.e., less evidence-based trials, less inclusion in school with peers).

**Methods:**

We implemented a novel model called the “Developmental and Sequenced One-to-One Educational Intervention” (DS1-EI) in 5- to 9-year-old children with co-occurring ASD and ID. The treatment protocol was adapted for school implementation by designing it using an educational agenda. The intervention was based on intensity, regular assessments, updating objectives, encouraging spontaneous communication, promoting skills through play with peers, supporting positive behaviors, providing supervision, capitalizing on teachers’ unique skills, and providing developmental and sequenced learning. Developmental learning implies that the focus of training is what is close to the developmental expectations given a child’s development in a specific domain. Sequenced learning means that the teacher changes the learning activities every 10–15 min to maintain the child’s attention in the context of an anticipated time agenda. We selected 11 French institutions in which we implemented the model in small classrooms. Each institution recruited participants per dyads matched by age, sex, and developmental quotient. Patients from each dyad were then randomized to a DS1-EI group or a Treatment as usual (TAU) group for 36 months. The primary variables – the Childhood Autism Rating scale (CARS) and the psychoeducational profile (PEP-3) – will be blindly assessed by independent raters at the 18-month and 36-month follow-up.

**Discussion and baseline description:**

We enrolled 75 participants: 38 were randomized to the DS1-EI and 37 to the TAU groups. At enrollment, we found no significant differences in participants’ characteristics between groups. As expected, exposure to school was the only significant difference [9.4 (±4.1) h/week in the DS1-EI group vs. 3.4 (±4.5) h/week in the TAU group, Student’s *t*-test, *t* = 5.83, *p* < 0.001].

**Ethics and dissemination:**

The protocol was authorized by the competent national regulatory authority (*Agence nationale de sécurité du médicament et des produits de santé*) and approved by the local Ethics Committee (*Comité de Protection des Personnes*) at the University Hospital Saint-Antoine (May 7, 2013). The findings will be disseminated through peer-reviewed journals and national and international conferences.

**Trial registration numbers:**

ANSM130282B-31 (April 16 2013) and ACTRN12616000592448 (May 6 2016).

## Background

Autism spectrum disorder (ASD) is characterized by the presence of atypical social communicative interaction and behaviors. The role of some genetic factors in ASD is known. However, there is a growing body of neurobiological research that indicates the presence of complex gene–environment interactions. Despite these findings, there is no approved biological treatment for this disorder and the first-line treatments pertain to psychosocial domains (1). Typically, ASD is diagnosed by means of a behavioral analysis during the 3- to 5-year-old age range; once diagnosed, the treatment is primarily delivered through behavioral interventions following different models. In essence, these models try to promote cognitive, communication, and behavioral skills that are considered essential to improve social skills in the long run (2, 3).

Several global interventions for core deficits in ASD have been proposed and assessed within clinical trials. The Treatment and Education of Autistic and Communication Handicapped Children (TEACCH) program uses many technical interventions to meet the individual needs of people with autism. The work program is tailored to some seminal aspects of ASD. First, it is centered on the individual. Individual needs are assessed through a comprehensive assessment of several developmental dimensions while taking into account emerging capacities. Second, it requires an understanding of autism, the adoption of appropriate adaptations and a broadly based intervention strategy (e.g., structured teaching, visual understanding, object manipulation, social communication skills) that builds on existing skills and interests. Third, the environment is organized to help children and adults understand and remember what to do (e.g., visual agendas, making expectations clear, and explicit, visual materials, structured architecture). The focus is on positive strategies to support behavioral and teaching strategies (4, 5).

Applied Behavioral Analysis (ABA) is a one-to-one intensive method that uses reinforcement of adaptative and acquired skills (6). The first structured attempts by Lovaas (7) were criticized (difficulties in generalization of learned behaviors; mechanical responses; lack of spontaneity) despite their encouraging first results. These criticisms led to the development of Pivotal Response Training [PRT], a more naturalistic behavioral treatment that has good documented effectiveness (8). PRT is a home-based intervention that includes parents in the routines. The method is based on choosing “pivotal” skills as the target of the treatment; following the child’s choice of activities and games; reinforcing not only the correct answer expected by the professional but also all (meaning complete or incomplete) forms of attempts to respond; alternating between acquisition and maintenance; and using intrinsic reinforcers.

The Early Start Denver Model (ESDM) is an early and intensive intervention approach for young children. The interventions are based on the following: (i) a curriculum that evaluates the child’s development across different developmental domains; (ii) specific procedures for learning and incorporating ABA principles, such as PRT; (iii) sessions focusing on interactions with children, interpersonal exchanges, and shared commitment with materials and activities of daily living; (iv) a positive affect, adults being responsive and sensitive to child cues; (v) verbal and non-verbal communication cues; (vi) proximal developmental windows, meaning that the focus of training is what is close to the developmental expectations given a child’s development in the according domain; and (vii) parents’ involvement. This program is implemented in small groups or individually at a specialized center or at home (3, 9).

The Developmental, Individual Differences, and Relationship-based (DIR) method is built on three axes: (i) the level of functional and emotional development reached by the child; (ii) the individual differences in information processing and motor planning; and (iii) the types of interactions that the child establishes with his/her partners (10). Floor Time is the core of the DIR method. It consists of sequences of guided play (15–20 min) that are repeated several times by parents throughout the day and are supervised by an expert. The DIR principles that should always be respected are to follow the child’s lead and support his/her initiative; to focus on joint attention; to close circles of communication; to create semi-structured problem solving; to contrast repetitiveness with playful obstruction; to support visual attention; and to work on imitation (10, 11).

In an attempt to capture the common components among these models and what could be learned from evidence-based studies, Narzisi and colleagues (12) delineated the following principles: the first group regards timing: (1) starting as early as possible; (2) minimizing the gap between diagnosis and treatment; (3) being intensive (not less than 3–4 h of treatment per day); the second group is based on viewing parents as partners and involving family; the third group gathers principles related to treatment program: (1) providing regular assessments, supervision and updating the goals of treatment; (2) encouraging spontaneous communication; (3) promoting skills through play with peers; (4) finalizing the acquisition of new skills and their generalization and maintenance in natural contexts; and (5) supporting positive behaviors rather than tackling challenging behaviors.

### Why Should We Implement a School-Based Intervention?

Despite the encouraging results presented earlier, most of those programs (a notable exception being TEACCH) do not target school-aged children and are not proposed to occur in a school setting. This is unfortunate because schools are a favorable location for autism interventions (13). Additionally, many children with ASD do not receive a sufficient amount of treatment (14), even in countries with free access to health care (15). Because children with ASD benefit from being with other peers at school, the gap between education research and education practice (16) may be a missed opportunity to offer more support to these children. Additionally, larger doses of treatment could be offered in school contexts, especially when interventions are administered 1:1 (17). Several agencies have recommended conducting interventions in school-based settings (18, 19). Two objectives should be combined: school-based core deficit interventions and school-based social communication practice (20). There are already several studies that have shown that school-based interventions are able to reach larger numbers of children with ASD. This may improve challenges with generalization by using learned skills regarding communication in a natural environment, such as in the classroom (21, 22). Additionally, the preschool context seems to offer opportunities to develop communication skills (23, 24), and by offering opportunities to enter into play groups, teachers can supply reinforcements of the non-verbal ASD child requests (25).

However, there are very few school-based social communication interventions, and in many cases, teachers at school do their best without guided specific interventions for ASD children in the classroom. Consequently, there is a lack of response from teachers to the communicative acts produced by children with ASD (26). General educational teachers provide infrequent verbal prompting with ASD children (27), and they more frequently engage in functional play than symbolic play (28). They also lack supervision (20). Thus, there is a paradox between the need for appropriate intensive interventions for ASD and what is proposed in most school settings. For example, Mudford and colleagues (29) showed that the implementation of an evidence-based ABA program in preschoolers was not complete: 93% of the participants were not provided the dose of treatment (40 h/week). Additionally, from an efficiency perspective, although several programs support the concept of tailoring interventions to the child’s needs and skills, to our knowledge, no one has questioned whether programs could be adapted according to teaching local skills.

### Why Should We Study Children with ASD and Intellectual Disability?

As expressed in the dimensional approach of the new classifications in the DSM-5 (30), intellectual disability (ID) is a frequent challenge and comorbidity in ASD. According to studies, ID co-occurs in 50 to 75% of ASD cases (31). Risk factors of comorbid ID in ASD are gender (despite the high number of males with ASD, the male/female ratio decreases in ASD comorbid with ID) and the existence of seizures or of a neurodevelopmental or genetic syndrome (32, 33). The co-occurrence of ID also appears to be a prognostic factor for long-term outcomes of ASD (12, 34) and a risk factor of the incidence of challenging behaviors that provoke severe morbidity in some cases (35). To date, very few models have specifically addressed ASD comorbid with ID, in particular when ID is in the severe-to-moderate range. Therefore, the need to focus on this understudied population is warranted. Here, we wonder whether or not children with comorbid ASD and severe ID may be receptive to a pedagogical content? For such children over 5 years, could an adapted and one-to-one cognitive program in school be a road to improve non-verbal and verbal communication and to promote social skills?

## Methods/Design

### Objectives

In this paper, our aims are to describe a school-based intervention program (a developmental and sequenced one-to-one educational intervention, DS1-EI) that was adapted to the French health and education system; to justify the principles that were followed to implement the method and adapt it to a low-functioning population (i.e., ASD comorbid with ID); to describe the randomized controlled trial we began; and to present the sociodemographic and clinical characteristics of the participants at baseline.

### Participants and Recruitment

**Figure 1:**
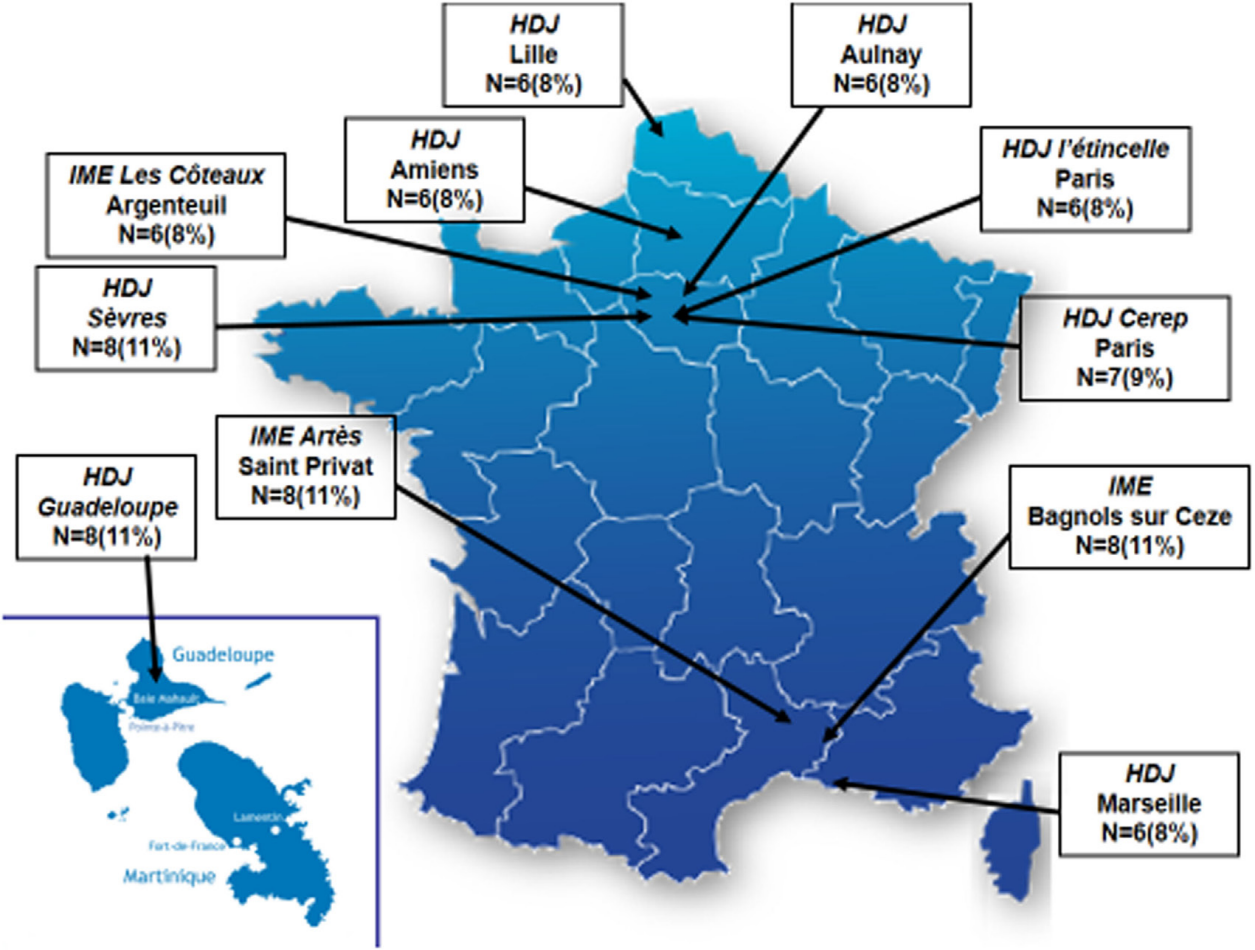
**Institutions and participants’ enrollment in the DS1-EI trial by site**. HDJ, *Hopital de Jour* (Day care medical center); IME, *Institut Medico-Educatif* (Special education center).

All participants were recruited in outpatient French health care institutions that are specialized in treating children with autism and intellectual handicaps. At the request of the French national health regulatory authority [*Agence nationale de sécurité du médicament et des produits de santé* (ANSM)] and the main sponsor [*Caisse Nationale de Solidarité pour l’Autonomie* (CNSA)], we balanced day care hospitals and special education clinics to have a representative sample of French institutions. In each institution, we obtained a specific commitment to accept the implementation of a DS1-EI school-based program as described below and to recruit the same number of participants to be randomized into a DS1-EI exposed group (called the DS1-EI group) or a treatment as usual (TAU) group who would serve as controls. The commitment also entailed having the required resources from local school authorities to implement the DS1-EI and to have the leading teacher from the classroom be supervised. To avoid bias in the TAU group as a result of the diversity of institutions, we decided to randomize participants by site. Figure 1 summarizes the list of institutions involved in the protocol and the number of patients by site. In total, we enrolled 75 participants.

Each parent provided informed written consent before inclusion. The inclusion criteria were a current diagnosis of ASD confirmed by a clinical assessment based on the International Classification of Diseases, 10th edition criteria and the Autism Diagnostic Interview-Revised (ADI-R) (36); an intellectual handicap with financial compensation from local agencies [*Maison Départementale du Handicap* (MDPH)]; being aged between 5 and 9 years; and having a communication developmental age of 24 months and under based on a Vineland assessment or a 3-year speech delay based on a Psycho-Educational Profile, third edition. We did not exclude children with known organic syndromes and/or non-stabilized neuropaediatric (e.g., seizures) or medical (e.g., diabetes mellitus) comorbidities. However, during the medical assessment, we specifically listed comorbidities. The exclusion criteria were limited to parents’ refusal to participate; family’s plans to change institutions in the short term for any reason; and patient’s severe behavioral impairments that would challenge treatment adherence. Of note, this last exclusion criterion was based on local institution staff decision. Before randomization, each site was requested to assess the IQ of the participants based on the Kaufman Assessment Battery for Children second edition (KABC-II) or, failing that, Vineland scores. Based on these results, dyads of participants matched for sex, age, and developmental quotient (DQ) were formed to limit the risk of bias between groups. Randomization before group allocation to TAU or DS1-EI group was performed by drawing lots in each dyad per site so that each site could have a TAU group and a DS1-EI group of three to four participants each. Randomization was performed by the methodological coordinating team at the Salpêtrière Hospital and was independent from local inclusion sites. TAU was defined as all therapeutic interventions given to a specific child. Given the study duration, we did not recommend not to change children’s therapeutic protocol in the TAU group during the study period. The trial duration was defined as 36 months but included 12-, 18-, and 24-month intermediate assessments.

### DS1-EI Treatment Principles

Participants randomized to the DS1-EI group received the specific experimental protocol four mornings per week (2 h 30 min); the rest of the week they continued to receive the usual protocol of each site. The treatment principles are summarized in Table 1. The setting was a small classroom with four pupils, but an adapted environment was proposed. Following the principles of TEACCH (5), each child was offered a desk, two chairs (one for the child, one for the adult working with the child), a screen where pictures of the child’s schedule and activities were provided, and a locker with his or her picture. In contrast to TEACCH, the child sat with his back close to the wall where the screen was placed (see pictures in the supplementary material S1). The setting also included a large table for mid-session group collaboration and a place offering benches and carpets where group participants (both children and adults) met at the beginning and ending of a session. Although the program did not reach the 40 h per week recommended by some programs (37), it remained intensive, with 10 h of DS1-EI plus other therapeutic practices according to each institution (e.g., occupational therapy; speech therapy; social skill group activities). The program followed developmental rules, meaning the training was focused on the nearest expected activity/skill of a given child’s development in a specific domain as recommended by the ESDM (9). In terms of timing, the program was sequenced in two ways. First, as in TEACCH, the 2 h 30 min sessions followed an anticipated and structured agenda that was presented for each child on a screen. When a novel activity started, the corresponding pictogram was shown on the child’s desk. Second, teachers were asked to change desk and activities every 10–15 min to maintain the child’s attention and to help him improve by challenging patient’s need of sameness. Thus, each 10–15 min, the child has a new activity and a new teacher. The program was also curriculum based and had specific educational objectives (see details below).

**Table 1:** **A developmental and sequenced one-to-one educational intervention (DS1-EI) for autism spectrum disorder: main principles**.

Characteristics	Brief definition	Justification
Setting	To be implemented in a small classroom with four pupils	TEACCH, Barton et al. (13)
	In an adapted environment	TEACCH
Intensive	One-to-one support 10 h per week in addition to other treatment practices (e.g., occupational therapy, speech therapy, psychotherapy)	ABA, ESDM
Developmental	The focus of training is what is close to the developmental expectation given a child’s development within a domain	ESDM
Sequenced	The 2 h 30 min sessions follow an anticipated and structured agenda	TEACCH
	Teachers change learning activities every 10–15 min to keep a child’s attention	Original
Curriculum based	A detailed assessment/curriculum is required to follow the developmental approach and to choose the appropriate cognitive/motor activity to be taught in each domain for preschoolers	ESDM, TEACCH
Educational objectives	Given the developmental quotient of the targeted children, the educational objectives are those of a second grade program for preschoolers (see Table 2 below for details)	French Ministry for National Education
Reinforcers	Supporting positive behaviors rather than tackling challenging behaviors	ABA, ESDM
	Using positive emotion engagement from teachers	ESDM
Group	Group activities are organized within the time schedule to encourage spontaneous communication and promote social skills through play with peers	Many programs
Supervision	Regular supervision of teachers with children’s objectives being updated	ESDM, ABA, DIR
Exploiting teachers’ unique skills	Implementation of the program will benefit from using teachers’ individual skills, such as their knowledge of a specific method (e.g., the use of Picture Exchange Program) or of a particular child	COMVOOR

*TEACCH, Treatment and Education of Autistic and Communication Handicapped Children; ABA, Applied Behavioral Analysis; ESDM, Early Start Denver Model; DIR, Developmental, Individual Differences and Relationship-based method; COMVOOR, Voorlopers in Communicatie*.

### Academic Training

Because DS1-EI was a program implemented in classrooms, both the curriculum and the objectives followed academic recommendations from the French Ministry of National Education. The curriculum was adapted from the French program for nursery and primary schools and *handiscol* principles (http://eduscol.education.fr). This was decided based on the idea that these recommendations were part of a teacher’s area of expertise and that it would promote participation in the program. Additionally, each classroom of *N* children was under the responsibility of one teacher helped by (*N* − 1) assistants, according to the 1-to-1 design of the program. In the same vein, one of the principles of the program was capitalizing on teachers’ individual skills. We believed that implementation of the program would benefit from using teachers’ specific knowledge (e.g., the use of the Picture Exchange Program). A detailed assessment/curriculum was a prerequisite of each child’s academic program because the DS1-EI was designed to follow a developmental approach, which required the selection of appropriate cognitive/motor activities for training within each domain. The curriculum is described in detail in the supplementary material S2. Regarding the academic/educational objectives, they were grouped into four domains: mathematics, language and communication, intermodality, and autonomy. Table 2 provides some examples of the activities by domain and level of child’s performance.

**Table 2:** **DS1-EI learning by domain and hierarchical proposals: examples of tasks**.

	Level 1	Level 2
**Domain 1: Mathematics**		
Numeration	Nursery rhyme to 5	Nursery rhyme to 39
Problem solving	Organizing stickers	Constructing a logical paradigm
**Domain 2: Language and communication**		
Communication	Improving joint attention	Asking for help
Oral language	Naming five objects	Making sentences to express a wish
Written language	Knowing letters from own name	Knowing all letters of classroom names
Graphics	Using sticks	Copying words with a model
**Domain 3: Intermodality**		
Writing/drawing and listening	Using various tools (paint, pastels, markers…)	Using different techniques (cut, paste, stencils…)
Musical activities	Imitating a rhythm	Learning songs with body movements
**Domain 4: Autonomy**		
Motor activities	Walking/Running/Swimming	Walking on a beam
Drawing activities	Using felt	Cut/Paste
Discovering the world	Describing a tree	Drawing a tree
Social skills	Waiting one’s turn	Improving autonomous work

### Teachers’ Training and Supervision

Each teacher and each assistant were trained by Annik Hubert-Barthelemy during a 1-week session. They were provided with a method presentation and were trained to use positive affect, shared engagement, responsiveness, and sensitivity to child cues, to focus on both verbal and non-verbal communication, and to support positive behaviors rather than tackle challenging behaviors. The DS1-EI detailed assessment/curriculum was explained, including how to keep learning proposals close to a given child’s developmental needs. The last 2 days of the training session was dedicated to define new objectives and adaptations. During the morning, the teacher with the help of his/her assistants had to fulfill children curriculums and to have related written observation. During the afternoon, curriculum was discussed and first learning activities for all domains were decided for each child.

Supervision was organized in three different steps: (i) daily sessions of verbal exchanges and written observations after the class about each child in each domain with all professionals (the teacher and the assistants); (ii) weekly supervisions by a psychologist; (iii) monthly supervisions by the main investigator to ensure the conformity of the program application and to help the teacher adapting the directives according to each child outcome.

### Primary and Secondary Variables

Table 3 summarizes the variables that we planned to measure at enrollment and at several time points throughout the trial. The primary outcome variables were (i) the Childhood Autism Rating scale (CARS), which measures autism severity (38); (ii) the psychoeducational profile, third edition (PEP-3), which measures the total DQ and 5-dimensional DQs related to cognition, receptive language, expressive language, fine motor skills, gross motor skills, and imitation, and (iii) the school’s assessment (39). Secondary variables included the following measures: (i) the *Vineland Adaptive Behavior Scale II* (VABS-II) as a behavioral scale of independence; this scale is assessed through a parent/educator interview and is used to assess the ability of children to perform the daily activities required for personal and social sufficiency. The VABS-II examines four specific domains: Communication, Daily Living Skills, Socialization, and Motor Skills. The subscale scores are totaled to yield an Adaptive Behavior Composite score (40). (ii) The KABC-II standardized neuropsychological assessment to measure intelligence skills. This battery measures Verbal, Performance, Working Memory, Processing Speed and Total quotients (41). (iii) The *Clinical Global Impression* (CGI), which was used to assess global severity (42). (iv) Finally, the *Children Global Assessment Scale* (CGAS) (43). To assess clinical change during the 36-month study, we used a single-blind procedure (independent raters were blind to study group allocation) for all clinical assessments (PEP-3, KABC-II, CARS). Blind assessment was not possible for the measures that required 2-week observations of the participants (CGI, CGAS, school tests) or a parental interview (ADI-R, VABS-II).

**Table 3:** **DS1-EI study procedure and evaluation criteria**.

	Selection	Inclusion	M12	M18	M24	M36
Inclusion criteria	X	–	–	–	–	–
Informed consent	X	–	–	–	–	–
CIM-10 diagnosis	–	X	–	X	–	X
Comorbidity	–	X	–	X	–	X
ADI-R	–	X	–	X	–	X
Vineland	–	X	X	X	X	X
CARS	–	X	–	X	–	X
CGAS	–	X	X	X	–	X
CGI	–	X	X	X	–	X
KABC	–	X	–	X	–	X
PEP-III	–	X	–	X	–	X
School assessment	–	X	X	X	X	X

*ADI-R, Autism Diagnostic Interview-Revised; CARS, Childhood Autism Rating Scale; CGI, Clinical Global Impression; CGAS, Clinical Global Assessment Score; PEP-3, Psychoeducational Profile, 3rd Edition; DQ, developmental quotient; KABC, Kaufmann Assessment Battery for Children*.

### Number of Participants

From previous studies that showed significant results in terms of efficacy it appears that the minimal number of patients in parallel design was 50 (2). This was the case for behavioral ABA approach [e.g., Ref. (6)] or for developmental ESDM approach [e.g., Ref. (3)]. The number of patients to enroll was based on the following theoretical statistics estimation: for a moderate effect size (α = 0.6), a power fixed at 80%, and a level of significance for a *p*-value fixed at <0.05, 80 patients randomized into two groups are required for a student *t*-test. Given our choice to use linear mixed models (see below) to take into account participant’s effect, we planned to recruit from 70 to 80 participants.

### Statistical Analysis

Statistical analyses will be performed using R Software, Version 2.12.2. To assess whether improvement occurs in both primary and secondary variables, we will use linear mixed models with change in a given variable explained by group exposure (DS1-EI vs. TAU), time (baseline vs. 18 vs. 36 months) and their interaction (group exposure × time). We will also include a random effect and a site effect. This should account for individual heterogeneity, site heterogeneity, variable scores at inclusion, and change specific to DS1-EI within the same statistical regression. For missing data when available, we will use the last observation carried forward. In case of a non-Gaussian distribution, we will study the log transformation (or other transformation when appropriate) to achieve a normal distribution. Lost or drop-out patients will also be compared between groups using a separate non-parametric comparison.

## Results

### Participants at t0 after Randomization

Table 4 summarizes the participants’ sociodemographic and clinical characteristics at enrollment. As expected, we found no significant differences at enrollment between the DS1-EI and TAU groups, indicating that the randomization by site did not introduce bias. As expected, exposure to school was the only significant difference found between the two groups: 9.4 (±4.1) hours per week in the DS1-EI group vs. 3.4 (±4.5) hours per week in the TAU group, Student’s *t*-test, *t* = 5.83, *p* < 0.001.

**Table 4:** **Sociodemographic and clinical characteristics of the participants after group randomization**.

	DS1-EI group (*N* = 38)	TAU group (*N* = 36)	Test, *p*
**Sociodemographics**			
Age, mean (± SD), year			
Male–Female	6.92 ± 1.57	7.34 ± 1.55	*t* = −1.15, *p* = *0.254*
Socioeconomic status	32 (84%)/6 (16%)	30 (83%)/6 (17%)	Fisher, *p* = *1*
**Clinical characteristics**			
ADI-R, current, mean (± SD)			
Social impairment score	21 ± 5.7	19.9 ± 5.8	*t* = 0.81, *p* = *0.423*
Communication score	11.8 ± 4	10.9 ± 3.2	*t* = 1.12, *p* = *0.266*
Repetitive interest score	6 ± 2.7	5.7 ± 3.1	*t* = 0.44, *p* = *0.654*
Developmental score	4.2 ± 0.8	3.9 ± 1.1	*t* = 1.29, *p* = *0.202*
CARS score	41.3 ± 7	40.2 ± 7.1	*t* = 0.67, *p* = *0.504*
CGI score	5.8 ± 1	5.7 ± 0.9	*t* = 0.44, *p* = *0.661*
CGAS score	27 ± 11.5	25.9 ± 11.1	*t* = 0.38, *p* = *0.705*
PEP-3 (all scores in DQ): mean ± SD			
Cognition	22.3 ± 11.1	23.4 ± 11.1	*t* = −0.44, *p* = *0.663*
Receptive language	14 ± 5.6	15.6 ± 8.6	*t* = 0.85, *p* = *0.4*
Expressive language	16.2 ± 5.8	16.8 ± 6.6	*t* = 0.4, *p* = *0.692*
Fine motor skills	26.6 ± 10.1	26.6 ± 9.7	*t* = 0.0, *p* = *0.99*
Gross motor skills	23.7 ± 8	24.8 ± 6.2	*t* = −0.62, *p* = *0.534*
Imitation	23.1 ± 7.8	25.8 ± 6.7	*t* = −1.57, *p* = *0.12*
Vineland (all scores in DQ): mean ± SD			
Communication	13.3 ± 8.2	14.2 ± 7.6	*t* = −0.49, *p* = *0.628*
Adaptation	26.5 ± 13.6	26.2 ± 12.5	*t* = 0.07, *p* = *0.94*
Socialization	13.7 ± 9	13.3 ± 9.4	*t* = 0.15, *p* = *0.88*
Daily living skills	30.9 ± 14.7	30 ± 12	*t* = 0.26, *p* = *0.79*
**Comorbidity**			
Intellectual disability level (< or > 40 using KABC)	27 (90%)/3 (10%)	26 (90%)/3 (10%)	Fisher, *p* = *1*
Known medical condition (no/yes)	34 (89%)/4 (11%)	27 (7589%)/9 (25%)	Fisher, *p* = *0.183*

*TAU, treatment as usual; ADI-R, Autism Diagnostic Interview-Revised; CARS, Childhood Autism Rating Scale; CGI, Clinical Global Impression; CGAS, Clinical Global Assessment Score; PEP-3, Psychoeducational Profile, 3rd Edition; DQ, developmental quotient; KABC, Kaufmann Assessment Battery for Children*.

## Discussion

We hope that the current trial will help demonstrate the feasibility of adapting and task-shifting a group of interventions used primarily as early interventions for autism to a school-based context and for use with older individuals with autism and intellectual disabilities. The school-based intervention program [the developmental and sequenced one-to-one educational intervention (DS1-EI)] was adapted from several methods including TEACCH (4, 5), ESDM (3), and ABA (6, 37). The key principles of the method are the intensity, the regular assessments and updating of objectives, the encouragement of spontaneous communication, promotion of skills through play with peers, support of positive behaviors instead of tackling challenging behaviors, regular team supervision, capitalization on teachers’ unique skills, and developmental and sequenced learning (2). The use of sequenced learning (i.e., teacher and activity change every 10–15 min to keep the child’s attention in the context of an anticipated time agenda) is likely the most original proposal. We are aware that treating children with autism and ID is very challenging. The effect sizes are typically small. To balance this risk of failure, we chose to have a rather long study duration with two single-blind assessments at 18 and 36 months. Given the number of sites, which may have introduced bias, we are satisfied that we did not find differences between the groups at baseline.

We are aware that there are several limitations to this trial. First, given the severity of the patients’ conditions, the study duration and the nature of the intervention, only a single-blind for the primary variables was feasible. Second, the method used to randomize participants (to limit the bias of TAU) combined with the study duration and the supervision of local teams is likely to modify local staff practice and to lead to some DS1-EI principles being used in the TAU group as well. Third, because our public sponsors required us to balance hospital/academic/large city sites and non-hospital/remote rural area sites, we cannot assume that we will achieve a uniform level of adherence to the program from one site to another. Finally, given the study duration, we do not know whether we will retain a sufficient number of patients in the program to maintain the statistical power needed for per protocol analyses.

## Declarations

### Ethics Approval and Consent to Participate

The current trial protocol was authorized by the competent national health regulatory authority [*Agence nationale de sécurité du médicament et des produits de santé* (ANSM)]. The trial registration number (ANSM 130282B-31) was obtained on April 16, 2013. The protocol was approved by the local Ethics Committee (*Comité de Protection des Personnes*) of the University Hospital Saint-Antoine on May 7, 2013. Also, it was registered on the Australian New Zealand Clinical Trial Registry for public information availability (ACTRN12616000592448). Potential participants received oral and accessible information about the study given participants’ cognitive profiles, and all parents/guardians were provided a written information leaflet about the trial. The information leaflet adhered to the current French guidelines for researchers on writing information sheets and consent forms. Only after written consent was obtained from the parents of potential participants did randomization occur.

### Consent for Publication

Persons’ data and images contained in this article are published with the consent of their parents/guardians.

### Trial Committees

A pilot study committee has been formed and includes the principal investigator of the trial and all local investigators and study collaborators. They will meet three times a year. A scientific study committee has also been established and includes two administrative members of the promotor (*La Croix Rouge Française*), four members of the pilot study committee and four other independent members (two professionals, two family representatives). They will meet once a year.

## Author Notes

Antoine Tanet is neuropsychologist at the Child and Adolescent Psychiatry department of the Pitié-Salpêtrière Hospital and PhD Student at IMI2S group (Institut des Systèmes Intelligents et Robotiques, CNRS, UMR 7222, Pierre et Marie Curie University Paris 6). Annick Hubert-Barthélémy is clinical psychologist and supervisor of the DSI-E1 program at the Croix Rouge Française. Graciela Crespin in clinical psychologist and head of the Programme de Recherche et d’Etudes sur l’Autisme. Nicolas Bodeau is a statistician at the Child and Adolescent Psychiatry department of the Pitié-Salpêtrière Hospital. David Cohen is Professor and head of the Department of Child and Adolescent Psychiatry at Pitié-Salpêtrière Hospital, and senior member of IMI2S group (Institut des Systèmes Intelligents et Robotiques, CNRS, UMR 7222, Pierre et Marie Curie University Paris 6). Catherine Saint-George is a child psychiatrist, member of IMI2S group (Institut des Systèmes Intelligents et Robotiques, CNRS, UMR 7222, Pierre et Marie Curie University Paris 6).

## Author Contributions

DC, CS-G, and AH-B designed the study; AH-B and GC created the treatment program; AH-B and AT implemented the program in each study site; NB created the data base for implementation of the program; DC and NB performed the statistical analysis; AT, CS-G and DC did a first version of the manuscript; all authors critically revised the final version of the manuscript.

## Conflict of Interest Statement

The authors declare that the research was conducted in the absence of any commercial or financial relationships that could be construed as a potential conflict of interest.

## Members of the GPIS (Groupe De Pédagogie Intensif Et Séquentiel) Study Group

Véronique Bur, Aude Brellier, Marie-Noëlle Clément, Christophe Chartier, Claire Ducateau, Jean-Louis Sarradet, Paris; Danièle Scellier, Hélène Petiton, Amiens; François Soumille, Marc Colombel, Marseille; Marc Bandelier, Louisa Garnil, Argenteuil; Emmanuel Damville, Isabelle Gylbert, Lille; Anne Juteau, Anne Vautrin, Aulnay-sous-Bois; Jean-François Havreng, Elisabeth Simonet, Sèvres; Georges Lançon, Yves Faure, Bagnols sur Cèze; Aurélie Broche, Myriam Garing, Saint Privat des Vieux; Carole Devaux, Sophie Michalak, Guadeloupe.
