# Supplementary material for: A Developmental and Sequenced One-to-One Educational Intervention for Autism Spectrum Disorder: A Randomized Single-Blind Controlled Trial
Source: Front Pediatr. 2016 Sep 26;4:99. doi: 10.3389/fped.2016.00099 (PMC5035746; doi:10.3389/fped.2016.00099)
Supplement: Supplementary file 1 [file presentation_1.pdf]

Developmental and Sequenced One-to-One Educational Intervention (DS1-EI): Examples of adapted environment and classroom setting

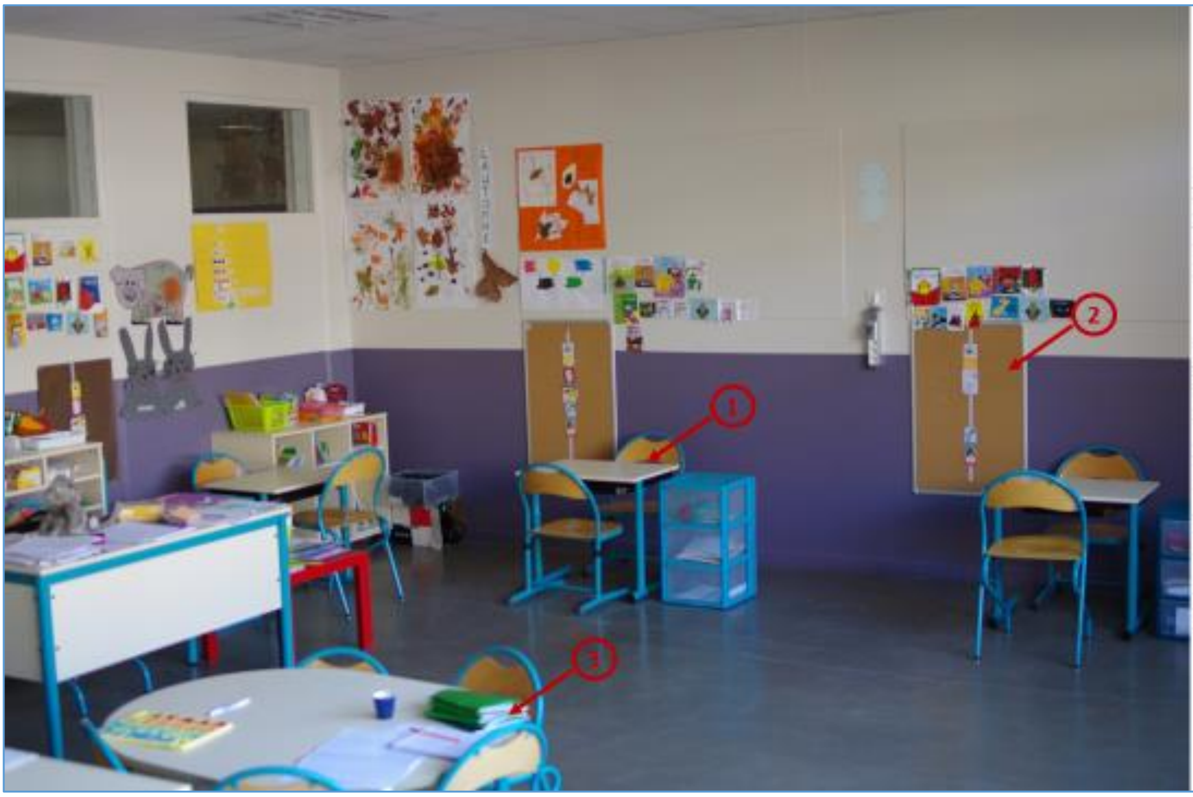

An example of one classroom with the DS1-EI setting

1. Child's and adult's desk and chairs
2. Child's screen with pictograms
3. The large table for mid-session group collaboration

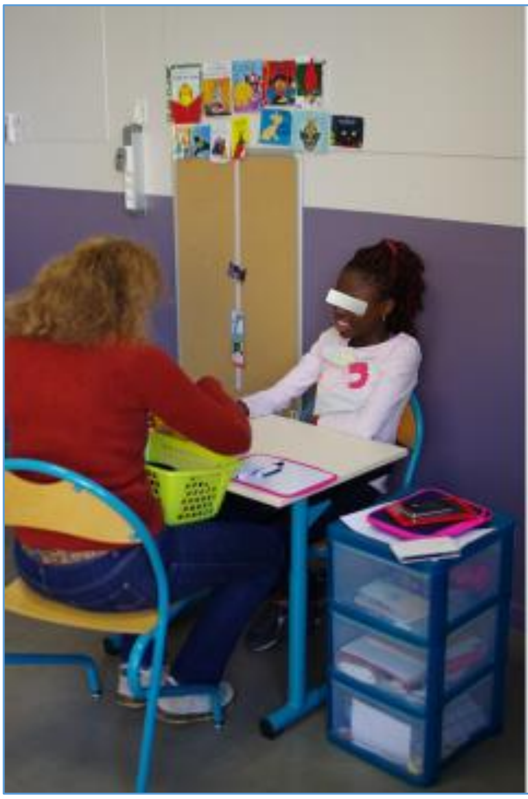

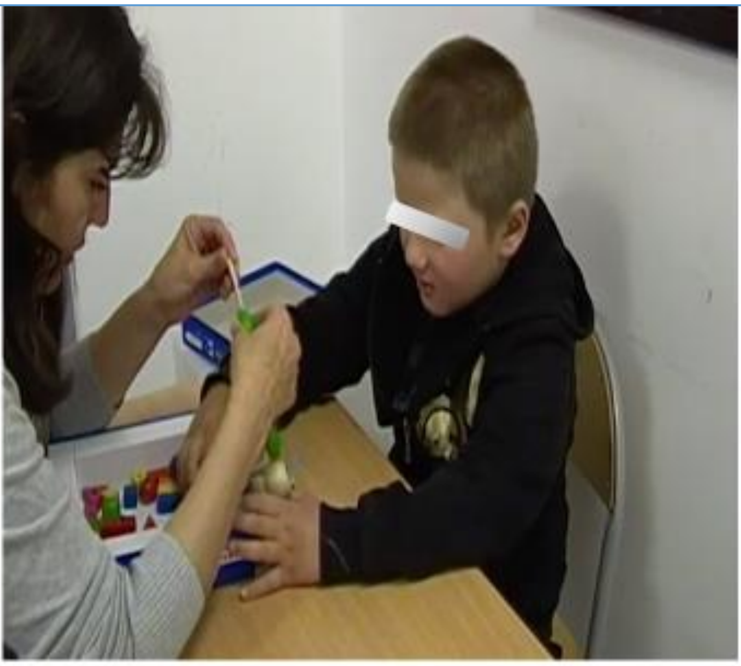

Each child is offered a desk, two chairs (one for the child, one for the adult working with the child). During the learning proposals, the child seats with his back close to the wall. The adult working with him seats in front of him.

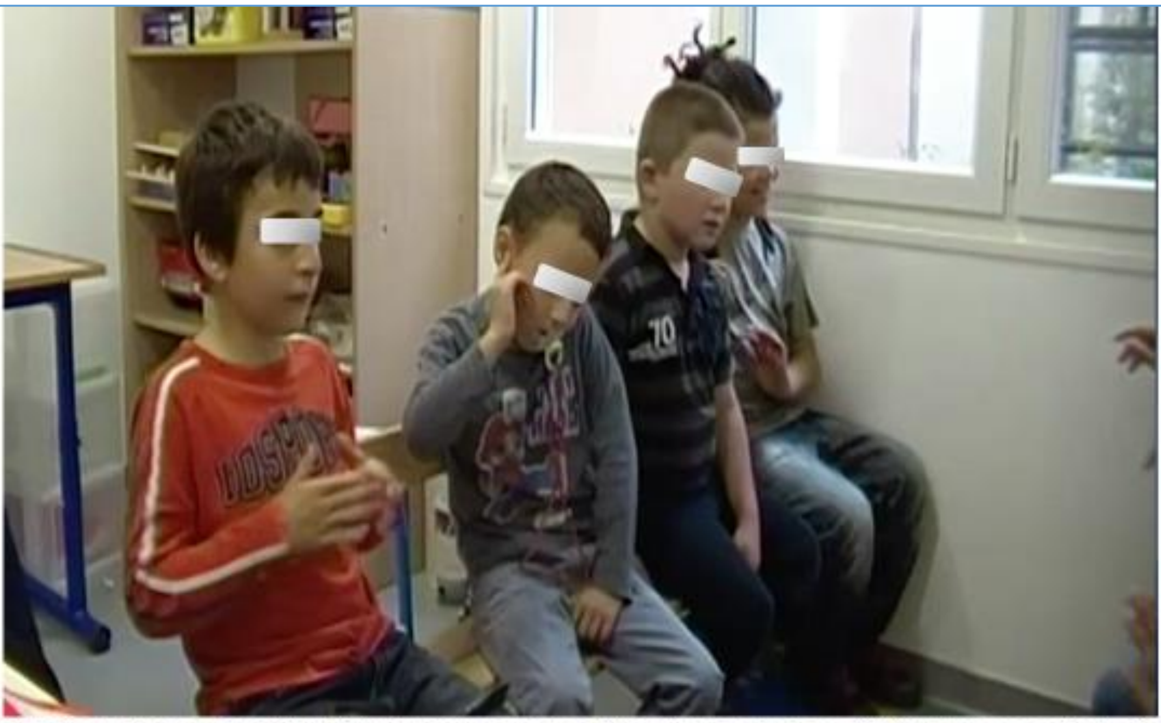

The setting also includes a large table for mid-session group collaboration and a place offering benches and carpets where group participants (both children and adults) meat at the beginning and ending of a session.

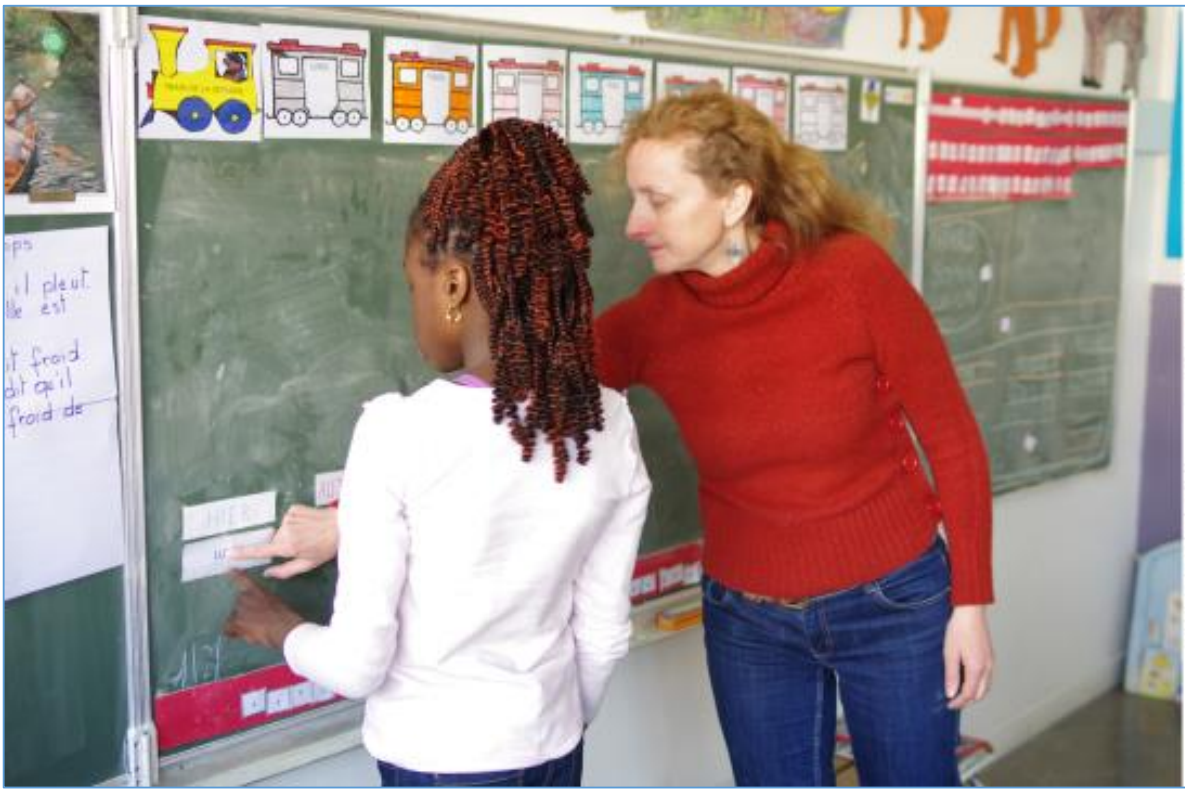

The teacher at the classroom board with a student working on time perception (today – *aujoud'hui*, yesterday – *hier)*

On the top, the train indicates the week days and their order. This is a common practice in *handiscol)*

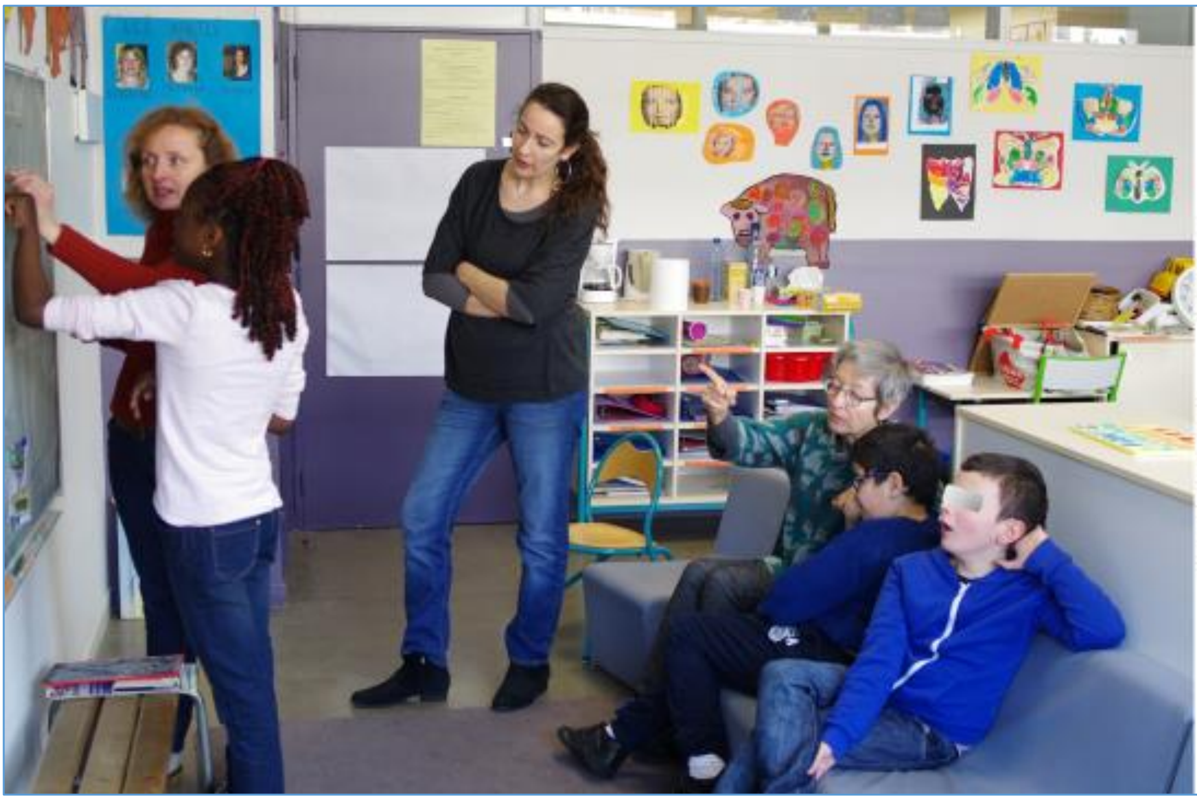

An example of group activities at the end of a session

One a the student is showing others her achievement and is encouraged by the group

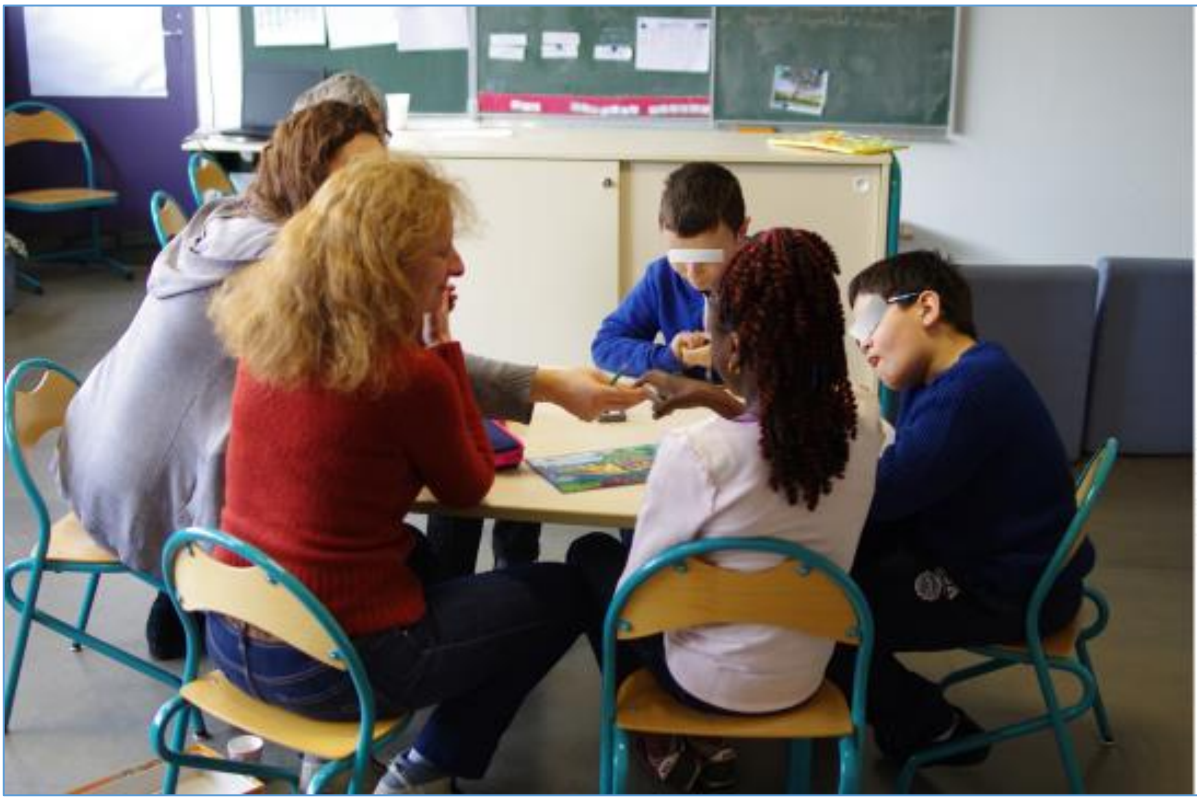

An example of group activities at the end of a session

All students, the teacher and the assistants are together around the group table
