# Supplementary material for: A Developmental and Sequenced One-to-One Educational Intervention for Autism Spectrum Disorder: A Randomized Single-Blind Controlled Trial
Source: Front Pediatr. 2016 Sep 26;4:99. doi: 10.3389/fped.2016.00099 (PMC5035746; doi:10.3389/fped.2016.00099)
Supplement: Supplementary file 2 [file presentation_2.pdf]

SUPPLEMENTARY MATERIAL 2

A Developmental and Sequenced One-to-One Educational Intervention (DS1-EI): Children's learning skills curriculum

Level 1: **Kindergarten-level evaluation for children with ASD**	Acquired	Being acquired	Not acquired
**Language mastery**			
**Oral language**			
Know the names of common objects (receptive language)			
Know the names of common objects (expressive language)			
Know the names of classmates			
Able to name people			
Imitate sounds			
Imitate words			
Use words relevantly			
Know the meaning of common guidelines			
Use sentences to express desires			
Use sentences to comment			
**Reading**			
Recognize the letters of one's name			
Recompose one's first name with a model			
Recompose one's name without a model			
Know the letters of one's name, name the letters			
Global reading of classmates' names			
**Graphics, fine manipulation**			
Use a glue stick			
Paste on defined areas			
Hold graphic and writing instruments properly			
Draw on horizontal lines			
Draw on vertical lines			
Draw on geometric lines: square, circle			
Draw on representative forms of everyday objects			
Colour in drawn shapes			
Be well-oriented to a sheet of paper			
Imitate gestures			
**Perception**			
Match objects by colour			
Pair two types of distinct objects			
Match pictures			
Paste stickers on specific points			
**Mathematics: Numbers**			
Know the digital rhyme to 5			
Spell out the rhyme up to 5 along with one's fingers			
Count a collection of objects up to 5			
Achieve a collection of objects up to 5			

**Mathematics: Logic**			
Reproduce a numeric line of stickers adhering to the direction of reading (left to right)			
Know the rhyme for weekdays			
**Discovery world**			
Know what a tree is			
Recognize a tree			
Name a tree			
Know the composition of a tree (trunk, leaves ...)			
Draw a tree			
**Music**			
Remember songs			
Perform songs with gestures and / or words			
Listen to excerpts			
Play a rhythm with an instrument			
**Visual arts**			
Respect the rules			
Use various tools (paint, pastels, markers ...)			
**Physical exercise and sport**			
Respect the rules			
Learn how to use your body to perform gymnastic moves (rolls ...)			
Move in different ways (run, crawl ...)			
Move on a beam			
Throw different materials in the right manner			
Take a gymnastics course			
Cooperate with a partner			
**Live together**			
Accept rules and instructions			
Share projects with adults			
Be well-oriented in the school space			
Be well-oriented in the rhythm of the day			
Wait your turn			
Work with another child while respecting the rules regarding noise, space and turns			
**Communication**			
Use words to communicate			
Use gestures to communicate			
Use pictures to communicate			
Understand instructions			
Express one's wills and needs			
Express joint attention			
Initiate interaction			

Level 2: **Preparatory course-level evaluation for children with ASD**	Acquired	Being acquired	Not acquired
**Language mastery**			
**Oral language**			
Know the names of common objects (receptive language)			
Know the names of common objects (expressive language)			
Use words wisely			
Know the meaning of common guidelines			
Use sentences to express desires			
Use sentences to express comments			
Say "yes" and "no" wisely			
**Reading**			
Recognize letters studied under different spellings (in script, uppercase and cursive) A, I, O, U, E, E, F, CH, L, S, T, V			
Know their names			
Know their sounds			
Read a syllable using known sounds			
Read two syllables combined			
Visually recognize identical syllables			
Recognize identical syllables aurally			
Identify syllables in a word			
Compare words letter by letter			
Find a word in a pattern in one graph			
Find a word in a pattern in different spellings			
Read words			
Read sentences			
Understand the meaning of reading			
Generally recognize words			
**Writing**			
Copy capitalized words with a model			
Copy words in cursive with a model			
Write words following letters in the lines of a notebook			
Write dictation, simple syllables			
Write dictation, combined syllables			
Write words globally without a model			
**Graphics and fine manipulation**			
Reproduce different graphics depending on the model			
Hold graphic and writing instruments properly			
Cut following lines, curves ...			
Paste forms and drawings following a model			
Make clean and neat work			
**Mathematics: Numbers**			
Know the digital rhyme to 39			
Write in encrypted numbers up to 39			
Read numbers up to 39 encrypted in writing			

Recognize numbers to 10 in literal writing			
Connect numbers called orally, in encrypted writing, in current settings (fingers, constellations of the dice) and in collections of objects			
Count up to 39 collections			
Achieve up to 39 collections			
Know the logical order of numbers (give the previous and the following numbers)			
Make a complete collection (adding elements)			
Adjust a collection by removing elements			
**Mathematics: Logic**			
Reproduce a logical reasoning			
Constitute a logical reasoning			
See drawn images and recognize certain characters			
Know whether an element has a certain character			
Read and understand a table offering different instructions			
**Discovery world**			
Know what a tree is			
Recognize a tree			
Appoint a tree			
Know the composition of a tree			
Draw a tree			
**Music**			
Remember songs			
Perform songs with gestures and / or words			
Listen to excerpts			
Play a rhythm with instruments			
**Visual arts**			
Respect the rules			
Use various tools (paint, pastels, markers ...)			
Using different techniques (cut, paste, stencils …)			
Have a personal project			
**Physical education and sport**			
Respect the rules			
Move from one point to another in different ways			
Move on a beam			
Launch different materials in the appropriate way (ball, javelin …)			
Take a gymnastics course			
Cooperate with a partner (adult or child) to follow a game rule			
Learn how to use your body to perform gymnastic moves (rolls ...)			
**Live together**			

Accept rules and instructions			
Share projects with adults			
Get used to the school space			
Wait your turn			
Work with another child respecting their space and turns while staying calm			
Work independently			
**Communication**			
Use different tools to communicate (gestures, words, pictures)			
Understand instructions			
Express desires and needs			
Ask for help			
Express joint attention			
Initiate interaction			
